# Has the DOTS Strategy Improved Case Finding or Treatment Success? An Empirical Assessment

**DOI:** 10.1371/journal.pone.0001721

**Published:** 2008-03-05

**Authors:** Ziad Obermeyer, Jesse Abbott-Klafter, Christopher J. L. Murray

**Affiliations:** 1 Harvard Medical School, Boston, Massachusetts, United States of America; 2 Institute for Health Metrics and Evaluation, University of Washington, Seattle, Washington, United States of America; 3 University of California San Francisco School of Medicine, San Francisco, California, United States of America; Harvard Medical School, United States of America

## Abstract

**Background:**

Nearly fifteen years after the start of WHO's DOTS strategy, tuberculosis remains a major global health problem. Given the lack of empirical evidence that DOTS reduces tuberculosis burden, considerable debate has arisen about its place in the future of global tuberculosis control efforts. An independent evaluation of DOTS, one of the most widely-implemented and longest-running interventions in global health, is a prerequisite for meaningful improvements to tuberculosis control efforts, including WHO's new Stop TB Strategy. We investigate the impact of the expansion of the DOTS strategy on tuberculosis case finding and treatment success, using only empirical data.

**Methods and Findings:**

We study the effect of DOTS using time-series cross-sectional methods. We first estimate the impact of DOTS expansion on case detection, using reported case notification data and controlling for other determinants of change in notifications, including HIV prevalence, GDP, and country-specific effects. We then estimate the effect of DOTS expansion on treatment success. DOTS programme variables had no statistically significant impact on case detection in a wide range of models and specifications. DOTS population coverage had a significant effect on overall treatment success rates, such that countries with full DOTS coverage benefit from at least an 18% increase in treatment success (95% CI: 5–31%).

**Conclusions:**

The DOTS technical package improved overall treatment success. By contrast, DOTS expansion had no effect on case detection. This finding is less optimistic than previous analyses. Better epidemiological and programme data would facilitate future monitoring and evaluation efforts.

## Introduction

After two decades of neglect by the international community, tuberculosis was again recognised as a major global health problem in the late 1980s.[1] The most recent global burden of disease figures[2] show that tuberculosis makes up 2.6 percent of burden in middle- and low-income countries, making it the ninth leading cause of death and disability worldwide; this estimate does not include tuberculosis in the context of HIV infection. The World Health Organization (WHO) estimates that 1.6 million people died of tuberculosis in 2005, of whom 190 000 were HIV-positive.[3] In part because of the powerful interaction between HIV and tuberculosis, global tuberculosis incidence is estimated to be rising.[4] Surveillance data suggest that multi-drug resistance (MDR) is significant in several countries with the highest burden of tuberculosis,[5] and recent reports of highly lethal strains of extensively drug-resistant tuberculosis[6] highlight the potential threat of expanding resistance.

WHO's tuberculosis control strategy has its roots in the now classic demonstration by Styblo in the 1980s that high treatment success rates were achievable in low-income settings from sub-Saharan Africa to Latin America.[7] Styblo's approach was adopted by the World Health Assembly in 1991 and renamed the DOTS (Directly Observed Therapy, Short-course) Strategy in 1994. The DOTS strategy has four key technical pillars: detection of smear-positive pulmonary tuberculosis using sputum microscopy, in patients presenting themselves to public clinics; directly-observed treatment with short-course chemotherapy; guaranteed continuous drug supply; and a case recording system tracking treatment outcomes. Early 2006 saw the launch of WHO's updated Stop TB strategy, designed to address three major challenges in tuberculosis control: continuing DOTS expansion, dealing with emerging types of tuberculosis like HIV-TB and MDR-TB, and engaging the broader health system including the private sector[8]; however, the four technical pillars of DOTS remain the “cornerstone” of the revised approach.[3]

The DOTS strategy has been adopted by 187 of 193 WHO member states at high levels of population coverage: WHO estimates that 89 percent of the world's population were living in areas implementing DOTS by the end of 2005.[3] While such administrative data do not necessarily reflect the proportion of all tuberculosis cases detected or the realities of patient access to care in developing countries, DOTS remains at the policy level one of the most widely-implemented and longest-running global health interventions in history. Given that DOTS will likely continue to occupy a central place in global tuberculosis control efforts in coming years, the question of what DOTS has or has not accomplished over the past 15 years is a central technical question; it is also critical to global health transparency and accountability.

WHO has evaluated the performance of DOTS in the course of specific studies[9]–[11] as well as detailed annual reports since 1997.[3], [12]–[21] These publications make use of two major indicators of programme performance: treatment results among patient cohorts and percent of incident tuberculosis cases detected by national programmes. The former indicator is based on real data collected in countries. The latter is difficult to study empirically given the paucity of data on the incidence of clinical tuberculosis, which forms the denominator of the case detection rate. A recent review concluded that only 10 sputum prevalence surveys exist as inputs to WHO's estimates of incidence for 211 countries and territories; of these only 4 were deemed rigorous, representative, and recent enough to provide useful information,[22] though a recent study[23] meeting the review criteria would bring the total to five. In a further 64 high-income countries with high-quality vital registration and surveillance systems,[24] it may be possible to assume that case notifications approximate incidence,[25] leaving 142 countries and territories without any credible information. WHO's estimation strategy for the these areas, which has been described in detail elsewhere,[26] is to directly guess the case detection rate on the basis of expert opinion. This number is then translated into an estimated incidence rate by taking advantage of the definition of the case detection rate: case notification rate over incidence rate. This circular strategy, in which guesses about programme performance feed into metrics used to evaluate programme performance, makes rigorous monitoring and evaluation difficult.

Given the difficulties of evaluating DOTS globally based on non-empirical estimates of its performance or flawed data, a limited number of studies have examined performance at country level.[27]–[30] These have shown mixed results, and those showing positive impact are weakened by methodological problems. First, they do not control for known determinants of tuberculosis incidence, notably HIV infection and socioeconomic development. Second, some studies make the questionable assumption that case notifications are an unbiased measure of incidence. Third, they do not differentiate the impact of increased financing from the technical elements of DOTS. Given the lack of firm evidence on the effectiveness of DOTS, many commentators have started to question some of the most fundamental principles of the strategy, most notably its effectiveness in case detection; its applicability in high-HIV, MDR, and resource-poor settings; and the validity of direct observation.[9], [31]–[43]

In this paper, we investigate the impact of the adoption of the DOTS strategy on case detection and treatment, the two goals of tuberculosis control programs. Like previous studies, our starting point is data on case notifications and reported outcomes of case treatment. Our approach differs from other efforts, however, in four ways. First, we restrict our analysis to data measured in countries, rather than *a priori* expert opinions with limited empirical inputs. Second, we use accepted methods of statistical inference to test the impact of DOTS expansion. Third, we identify and address known biases and distortions in the empirical record. Fourth, our evaluation is independent, in that none of the authors of this study has a professional or financial stake in the success of the DOTS strategy. This avoids the conflict of interest that can arise when organizations assess their own performance. We build on previous studies of the effectiveness of global health interventions using similar methods.[44], [45] This evaluation may have significant implications for the future of the Stop TB strategy, allowing policy planners to correctly identify priority areas for strategic and operational improvement in light of the accomplishments of the past 15 years. It may also inform thinking on a range of emerging health interventions that require case detection and delivery of life-long treatment, from medications for diabetes and cardiovascular disease[46] to antiretrovirals for AIDS.[47]

## Methods

We seek to analyse the impact of the DOTS strategy on two key dimensions of tuberculosis control: case detection rate (CDR), defined as the ratio of notified to incident cases, and treatment success rate (TSR), defined as the sum of the fraction of cases completing treatment and the fraction converting from smear-positive to negative. Direct assessment of the impact of DOTS on case detection would require data on tuberculosis incidence, which is unknown. Our analytic method takes advantage of the relationship between case notification rate (the ratio of notified cases to population), and case detection rate: changes in notifications are driven by changes in detection or changes in incidence. Our model (described in more detail in Technical Appendix S1) analyzes notifications as a function of epidemiological correlates of tuberculosis as well as programmatic variables. This analytically isolates determinants of incidence from other drivers of change in notifications, so that any remaining change in notification rate becomes attributable to change in case detection. Since the definition of a smear-positive case is largely consistent across time and place, and since smear-positive cases have been the primary target of the DOTS strategy since its inception,[3] we focus our analysis on smear-positive notification rate (SSNR).

In general, only omitted determinants of case notifications that are correlated with changes in programmes would introduce bias, while determinants uncorrelated with programme changes would not. For example, changes in the way cases are reported to WHO can also produce changes in the case notification rate. If these changes are non-random, for example if DOTS expansion improves tuberculosis reporting systems without affecting true detection rate, our results could be biased in favour of detecting a programme effect. We thus perform a literature review to identify countries with evidence of changes in reporting modality for tuberculosis cases, for example changes in case definitions or introduction of electronic or Internet-based technologies, over the time period of our analysis.

In order to capture determinants of variation in incidence, we include two main variables. First, we include country-level estimates of HIV population seroprevalence,[48] constructed by UNAIDS on the basis of empirical data from surveillance sites and population-based surveys;[49] this restricts our analysis to 121 developing countries for which both HIV estimates and DOTS programme data are available, including 21 of 22 “high-burden” countries.[3] HIV prevalence is lagged by five years to account for the period during which significant immune compromise accelerates breakdown from latent tuberculosis infection to clinical disease. Second, GDP per head is included to control for the well-known relationship between tuberculosis incidence and socio-economic status.[50], [51] We also test other variables that may contribute to or correlate with changes in tuberculosis incidence: smoking impact ratio,[52] apparent cigarette consumption,[53] population age structure, urbanization, total years of schooling, and literacy rates.[54] Finally, we also test factors unrelated to DOTS expansion that may contribute to increased case detection: national health expenditure per head, and estimated coverage levels of diphtheria, tetanus, and pertussis vaccination (DTP3).[55] Including the latter is based on the assumption that coverage of a basic health intervention measures general health system coverage, with the goal of isolating the impact of DOTS from that of health system change.

Our major indicator of DOTS expansion is DOTS population coverage (DPC), defined and reported by WHO as the percentage of national population living in areas (*e.g*., districts, counties) implementing DOTS. Population coverage is used by WHO as the primary measure of DOTS expansion, along with estimated case detection rate. To further confirm the validity of this measure, we explore the relationship between population coverage and percent of all cases notified under DOTS in countries, and find a significant positive relationship (univariate OLS: *R^2^* = 0.78, *β* = 0.88, *p*<0.0001). We use this indicator as a measure of DOTS expansion at the administrative level. We also use DOTS treatment success rate as an independent variable, based on the assumption that higher success rates indicate higher programme quality on the ground. Because population coverage and treatment success both measure programme delivery, we test one variable at a time rather than both in the same model.

We next seek to analyse the impact of DOTS expansion on overall treatment success rate; this is only available for smear-positives. Countries report treatment outcomes separately for DOTS and non-DOTS cohorts, but the dataset contains many missing values (41 percent of all country-years from 1995 to 2005 for DOTS, 79 for non-DOTS). To test the impact of adoption of the DOTS strategy on overall treatment success, we restrict the analysis to country-years where treatment success is reported for both DOTS and non-DOTS cases. We calculate mean treatment success rate (DOTS and non-DOTS, weighted by number of cases) and model it as a function of DOTS population coverage, controlling for general development by using GDP per head. We perform the analysis both with and without HIV seroprevalence, to control for the possibility of poorer outcomes in HIV-positive patients.[56]–[58]

We construct all models using standard time-series cross-sectional methods[59], [60] with a lagged dependent variable[61] and country fixed effects (*i.e.*, dummy variables)[62] to capture the effect of omitted variables and isolate changes produced by DOTS programme variables. Fixed effects are used to capture differences in incidence, case detection, and determinants of treatment success that are specific to individual countries and invariant over the period of observation; in other words, they are by definition unrelated to the rollout of the global DOTS strategy. Since our dataset contains multiple observations from the same country over time, there is the risk of non-independence in standard errors; we correct for this by clustering standard errors by country.[63] Technical Appendix S1 contains more details regarding our data sources and methods, as well as supplemental models not presented in the main text.

## Results

**Figure 1:**
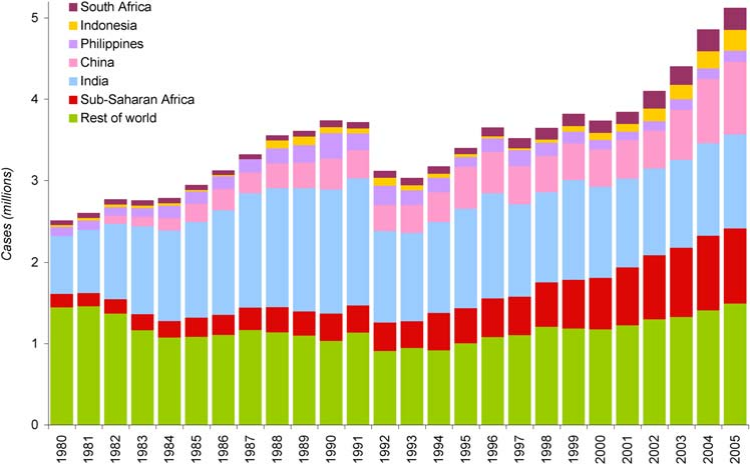
Total smear-positive and smear-negative tuberculosis cases notified to WHO, 1980–2005

Figure 1 shows the total number of tuberculosis cases of all forms reported to WHO since 1980. Any changes in the number of cases reported may be due to changes in six factors: 1) case detection rate, 2) incidence rate, 3) population size, 4) case definitions, 5) proportion of detected cases that are recorded at the local level, and 6) proportion of cases recorded in the periphery that are reported to the central government and to WHO. Our analysis seeks to minimise confounding referable to factors other than changes in incidence and case detection rate. Since the latter four factors are either measurable or to some extent predictable, they are addressed in the analysis. The effect of population growth, for example, can be removed by using case notification rate (CNR): dividing notifications by population, we calculate that global case notification rate varied between 57 and 71 per 100 000 population from 1980 to 2003; they surpassed 70 per 100,000 in 1990 and again in 2004, then increased to 79 per 100 000 in 2005. Evolving definitions of smear-positive cases are likely responsible for the fluctuation in case numbers seen in the early 1990s; we thus restrict our analyses to the period from 1995–2005, when consistent data are available.

**Figure 2:**
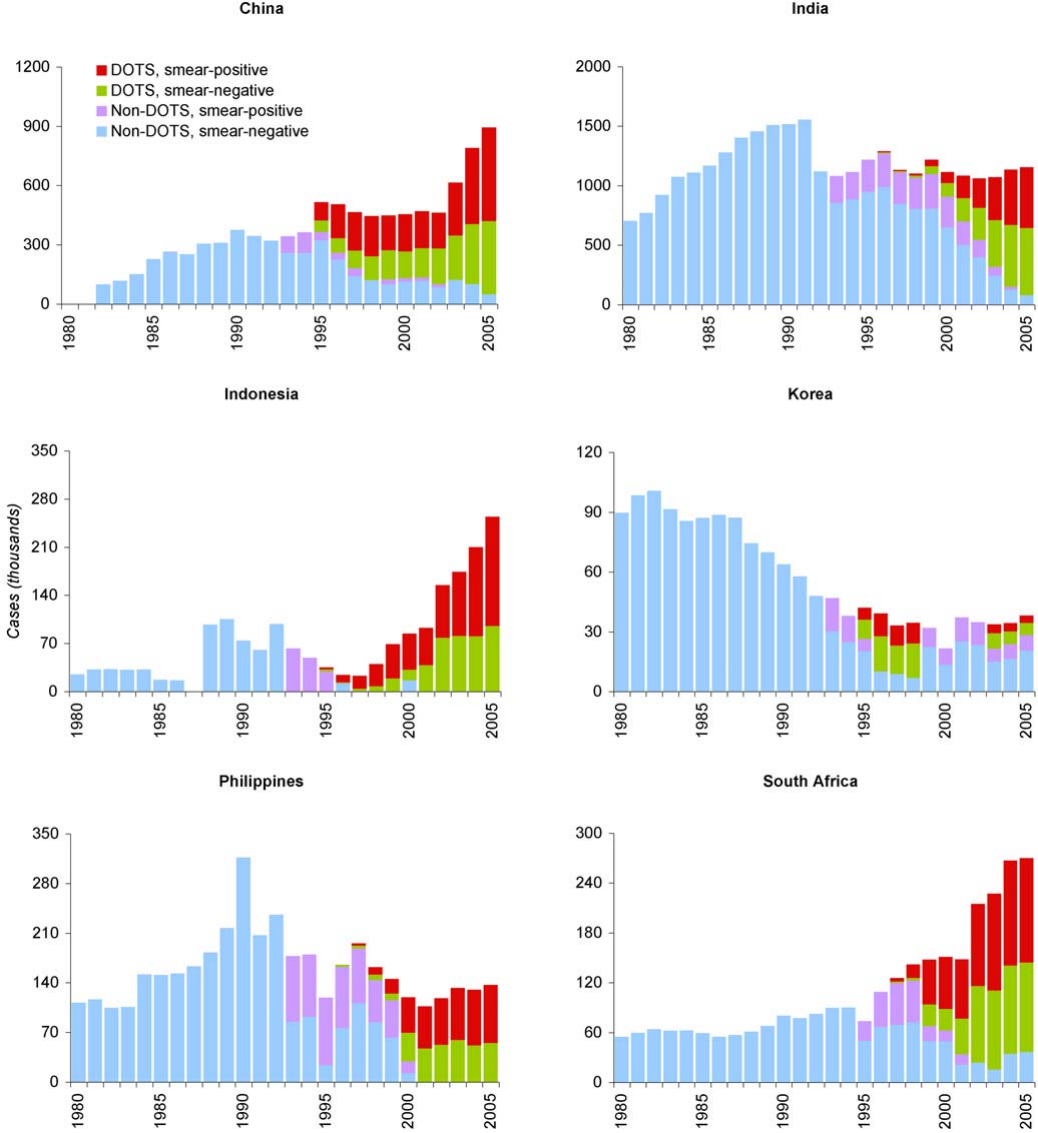
Tuberculosis cases reported to WHO in six of ten countries with known recent transitions to electronic case recording or reporting, 1980–2005

Improvements in recording and reporting technology are more difficult, but equally necessary, to consider. Results of a literature review indicate that at least ten countries transitioned fully or partially to electronic reporting systems over the period of our analysis: Botswana, China, India, Indonesia, Korea, Lesotho, Namibia, Nepal, Philippines, and South Africa.[3], [20], [21], [64]–[69] Such changes can distort case notification trends by increasing the number of cases reported to the centre without affecting case detection on the ground, complicating interpretation of time trends. Figure 2 shows trends in notified cases for six of these countries, illustrating discontinuities in number and composition of reported cases. Since these may be referable to arbitrary changes in reporting modality rather than sudden changes in incidence or detection, we undertake all subsequent analyses both with and without these ten countries.

**Figure 3:**
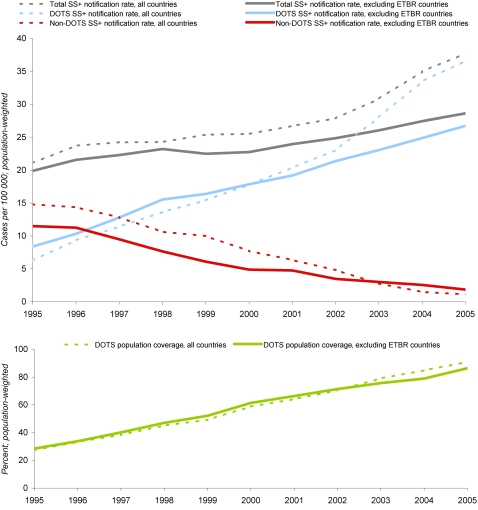
Smear-positive tuberculosis notifications, shown alongside DOTS coverage, 1995–2005; Figure 3a (top): Smear-positive tuberculosis notifications to WHO, as reported vs excluding countries with known transitions to electronic tuberculosis reporting (ETBR: Botswana, China, India, Indonesia, Korea, Lesotho, Namibia, Nepal, Philippines, South Africa), 1995–2005; Figure 3b (bottom): Percent of world population living in areas (e.g., districts, counties) implementing DOTS, 1995–2005

Figure 3 presents the trend in the number of smear-positive cases notified to WHO from 1995 to 2005 alongside changes in DOTS population coverage, with and without countries that adopted electronic reporting over the period of analysis. When these countries are removed, the sharp upturn since 2003 disappears and smear-positive notifications increase steadily from 20 per 100 000 in 1995 to 29 per 100 000 in 2005, 43 percent in ten years. The composition of the total smear-positive cases detected has shifted dramatically from non-DOTS to DOTS.

The four models in Table 1 attempt to characterise the relative contribution of changes in tuberculosis incidence and changes in case detection rate, including the impact of DOTS programmes, on smear-positive notifications. In all models, the one-year lagged smear-positive notification rate is significant, confirming the presence of serial autocorrelation (Wooldridge test, *p*<0.0001). GDP per head has no significant effect on notifications, and HIV seroprevalence is significant only in models with treatment success as the programme variable. Neither DOTS population coverage nor treatment success rate has a statistically significant relationship on the case notification rate in these models, or a wide range of alternative specifications and analyses. Repeating our analysis on notification rates for all forms of tuberculosis likewise shows no DOTS effect. Increasing urbanization significantly raises notification rates, but including this variable has no impact on the DOTS coefficients. Technical Appendix S1 Tables S1, S2, S3 contain results from additional models.

**Table 1:** Smear-positive notification rate as a function of GDP, HIV, and DOTS programme variables, 1995–2005

		*All countries*		*Excluding countries with electronic reporting*	
		*Model 1*	*Model 2*	*Model 1*	*Model 2*
**GDP per head,**	*Coefficient*	0.008	0.006	0.005	−0.004
**USD thousands**	*SE*	0.008	0.013	0.008	0.010
**HIV seroprevalence**	*Coefficient*	0.45	**1.40**	0.40	**2.63**
**(five-year lag)**	*SE*	0.52	0.53	1.01	1.11
**DOTS population coverage fraction**	*Coefficient*	0.05	-	0.04	-
	*SE*	0.05		0.05	
**DOTS treatment success fraction**	*Coefficient*	-	0.04	-	0.02
	*SE*		0.12		0.13
**Lag of SSNR**	*Coefficient*	**0.56**	**0.49**	**0.55**	**0.47**
**(one year)**	*SE*	0.05	0.05	0.05	0.04
**Constant**	*Coefficient*	**0.69**	**1.14**	**0.82**	**1.23**
	*SE*	0.27	0.14	0.28	0.35
***Observations (country-years)***		1128	887	1024	792
***R^2^***		0.95	0.96	0.95	0.95

Coefficients significant at the 0.05 level are in bold. All standard errors are clustered by country.

* Excluded: Botswana, China, India, Indonesia, Korea, Lesotho, Namibia, Nepal, Philippines, S Africa

*(Independent programme variable: Model 1*—*DOTS population coverage, Model 2—DOTS treatment success rate)*

**Figure 4:**
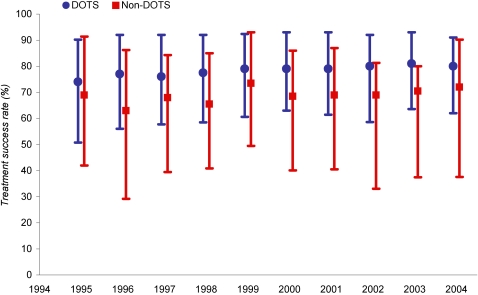
Median and 10–90th percentile range of treatment success rates for countries reporting DOTS and non-DOTS treatment outcomes, 1995–2004

The magnitude of total change in overall treatment success rate as a result of DOTS expansion is unknown, since reporting on treatment outcomes in non-DOTS programmes is scattered and often inconsistent. In order to rigorously assess the impact of DOTS on the treatment success rate, we examine countries that report in the same year treatment success data for both DOTS and non-DOTS smear-positive cases. The available data on DOTS and non-DOTS treatment success in these countries are summarised in Figure 4. The difference in the median treatment success rate over this period ranged from 5 to 14%.

While DOTS treatment success rate is consistently higher, this could reflect a correlation rather than a direct effect of DOTS expansion on mean country treatment success. Because of serial autocorrelation (Wooldridge test, *p*<0·0001) we use methods requiring data on the concurrent and prior-year success rates, which restricts our analysis to under 200 country-years. Table 2 summarises the results for two models, with and without HIV. GDP per head and HIV seroprevalence are non-significant. DOTS population coverage is statistically significant in both models for this limited dataset. The DPC coefficient in the model including HIV indicates that expanding DOTS coverage from zero to 100 percent of the population raises mean treatment success rate by 18 percent (95% confidence interval: 5–31%). Given the small sample size and the fact that the coefficient on the lagged success rate was non-significant, we also repeat the analysis without the lagged variable; this increases sample size by approximately 70 country-years, but does not alter the results (see Technical Appendix S1 Table S4).

**Table 2:** Mean national treatment success rate (DOTS and non-DOTS, weighted by cases) as a function of GDP, HIV, and DOTS population coverage, all countries, 1996–2004

		*Without HIV*	*With HIV*
**GDP per head,**	*Coefficient*	0.002	0.006
**USD thousands**	*SE*	0.009	0.014
**HIV seroprevalence**	*Coefficient*	-	−0.28
	*SE*		0.35
**DOTS population coverage fraction**	*Coefficient*	**0.16**	**0.18**
	*SE*	0.05	0.07
**Lag of TSR**	*Coefficient*	0.21	0.21
**(one year)**	*SE*	0.21	0.21
**Constant**	*Coefficient*	0.31	**0.64**
	*SE*	0.19	0.18
***Observations (country-years)*** *		191	159
***R^2^***		0.81	0.81

Coefficients significant at the 0.05 level are in bold. Standard errors are clustered by country.

* Model with HIV includes 45 countries, models without HIV include 55 countries; both include countries from all six WHO regions.

## Discussion

Despite the realities of limited data on tuberculosis, our analysis provides empirical evidence that the expansion of the DOTS strategy led to improved treatment success rates. This finding is significant in the setting of debate as to the actual impact of direct observation on treatment success, particularly health facility-based direct observation in countries with sparse health infrastructure.[40], [70]–[73] This analysis, however, cannot identify which element of the overall DOTS technical package has been effective in increasing treatment success rate. Given the literature on treatment outcomes in HIV-positive tuberculosis patients, we feel that the model incorporating HIV is the most credible, implying an increase of 18 percent with full DOTS coverage. This number may well be biased downward, due to the small sample of countries and to the possibility of selection effects: countries that consistently report non-DOTS treatment outcomes are likely to have better non-DOTS programmes than countries that do not. Given this potential bias, the results should be interpreted as the minimum effect of the DOTS strategy on treatment success rates.

In contrast, the analysis failed to detect any impact of national adoption of the DOTS strategy on case notifications, and by inference case detection rates, when controlling for determinants of tuberculosis incidence. This conclusion was robust across a wide range of specifications and functional forms. This finding is considerably less optimistic than recent reports, which imply that DOTS expansion has driven increases in estimated smear-positive case detection rate from approximately 40 percent in 1995 to 2000 to 60 percent in 2005—a 50 percent increase in five years.[3] As noted above, our technical approach differs from previous efforts in three ways: use of accepted statistical methods, correction for known biases, and use of empirical data. Given the discordance of our finding with previous studies, it is important to discuss the potential reasons that our time-series cross-sectional analysis may be flawed.

First, it is possible that these methods are not sufficiently sensitive to detect the impact of DOTS expansion on case notifications. Panel regression techniques, however, are widely used and accepted in the econometric and political science literature, and have been used to measure effects in a wide variety of fields from political economy to international relations.[74] In addition, the fact that these methods detect the effect of DOTS expansion on treatment success rate in all models argues against this interpretation.

Second, if we have omitted factors predicting declines in case notifications that are also correlated with DOTS coverage, our estimate of the impact of DOTS on case detection would be biased downward. One possibility would be that DOTS population coverage itself has an immediate negative effect on incidence, which exactly balances a positive DOTS effect on case detection rate. However, this possibility seems unlikely given the natural lags built into the epidemiology of tuberculosis: changes in risk of infection affect incidence of active cases only after a significant period of breakdown from latent infection to clinical disease, generally much longer than the one-year units of this analysis.[75], [76] We have been unable to identify other drivers of notifications that would be negatively correlated with DOTS population expansion. Indeed, unlike previous evaluations of DOTS, we explicitly control for several sources of bias in case detection trends. Variables measuring HIV seroprevalence, income per head, and time-invariant attributes of countries (*i.e.*, fixed effects) are included to isolate the effect of DOTS more precisely, and none would be expected to obscure the presence of a true effect. GDP was found to be non-significant in nearly all models, and HIV was only significant in some models; this may reflect the fact that GDP or HIV growth within countries over the study interval is small relative to the across-country differences, meaning that country dummy variables capture most of the effect. In the case of HIV, this may also indicate that case notifications are not growing at a rate commensurate with increasing TB-HIV.

Third, any analysis based on case notification data may be biased towards detecting a positive DOTS effect, as institution of DOTS may lead to improved reporting of cases already detected. Spurious trends due to reporting changes are ubiquitous in the case notification series. The massive increase in cases reported in China from 2003 to 2005 coincides with the introduction, in the wake of the SARS epidemic, of a mandatory Internet-based case reporting system for all infectious diseases, covering nearly all county hospitals and tuberculosis clinics, and a large fraction of village dispensaries.[21], [65] Since the start of DOTS implementation in China under the auspices of the World Bank, there has been widespread recognition that cases of tuberculosis diagnosed in general hospitals were under-reported: hospitals have a powerful financial incentive not to report cases to the tuberculosis programme, in order to maintain revenues from patients. The new surveillance laws and reporting system now make it difficult for hospitals not to report these cases. As a result, a certain number of cases, which would previously have been diagnosed and treated in hospitals but not reported to tuberculosis clinics, began to be centrally reported. There is no reason to believe that this represents a real increase in the number of cases detected. Similarly, the major increase in cases from 2002 to 2005 in South Africa coincides with the roll-out of an electronic case notification system[66], [77] in the country's largest provinces, which again reflects better recording of cases centrally rather than a true rise in the number of cases diagnosed. Clearly, reporting changes introduce distortions into case notification trends, and we chose to perform our analyses both with and without countries with known changes to minimize arbitrary changes in the series. While these countries account for a large percent of all tuberculosis cases (53 percent between 2001 and 2005), they represent only a small minority of the 121 national DOTS programmes that are the unit of this analysis. In any case, exclusion of these countries had no appreciable impact on results. Since WHO does not systematically report on changes in reporting modalities, it is impossible to exclude the possibility that reporting changes in other countries may be affecting our results; however, this would result in upward bias, not downward bias, and thus would not explain our finding that DOTS has no effect on case detection.

The analytical approach of this paper, using case notifications to detect the impact of DOTS on case detection by controlling for determinants of incidence, would not be necessary if real measures of tuberculosis epidemiology were available. The approach of WHO to date has been to strengthen routine tuberculosis surveillance systems in the developing world, such that as coverage approaches 100 percent, case notifications become a reasonable measure of incidence.[25] Given the failure of most countries to implement even basic vital registration systems after decades of effort,[78] it is clear that complete surveillance coverage is an ambitious goal. Better data on real trends in tuberculosis epidemiology are needed in the interim.

While there may in fact be an opportunity to track trends in tuberculosis by examining death rates in those countries with complete vital registration,[79] direct measures of tuberculosis incidence or prevalence remain the gold standard for epidemiological surveillance. At least eleven countries have undertaken serial population-based sputum prevalence surveys over the last 60 years,[23], [80] but given the substantial costs of this approach, there is little prospect that such surveys will provide a widespread basis for the frequent epidemiological measurements required for monitoring. For some countries, serial skin test surveys are available.[81] Interpretation of these, however, is legendarily difficult due to confounding from inconsistent definitions of infection,[82] environmental mycobacteria,[83], [84] ‘boosting’ from repeat testing,[85] BCG vaccination,[86], [87] and increasing selection bias in BCG scar-negative children. To make epidemiological surveys a viable tool in countries without the resources to implement prevalence surveys, new measurement strategies are clearly needed.

Pending the development of such methods, case notifications are a key indicator of programme performance. Thus any efforts to disentangle changes in reported case numbers—due for example to the adoption of new reporting technologies in China, South Africa, and elsewhere—from real trends in the number of new cases detected would represent a major contribution to monitoring performance. WHO currently presents increases in case notifications due to reporting changes as real increases in the case detection rate, rather than concluding that their previous estimates were artificially depressed by poor reporting.[21] Indeed, on the basis of the spurious trend resulting from China's new Internet-based reporting system, a major increase in global case detection has been claimed.[88]

There are three potential ways to explain why DOTS failed to increase case detection. First, the problem could be one of resource constraints. Including tuberculosis programme expenditures in the models would have allowed us to distinguish the impact of DOTS expansion from changes in funding levels, but comparable expenditure data are not available globally.[3] Second, current constraints of developing countries' health systems, including inadequate geographical and financial access to facilities or severe limitations of human resources, may be limiting the effectiveness of DOTS. The Stop TB strategy's emphasis on engaging health systems and the private sector is one potential solution to this problem. In poor rural areas of developing countries, however, where there are no private and few public providers, even the Stop TB strategy may not be sufficient. Finally, it is possible that the DOTS technical strategy itself limits the potential case detection rate. Though the DOTS strategy has ambitious targets for increasing detection, the emphasis on ‘passive’ case finding means that there is no provision in the key components of the strategy that specifically pertains to finding new cases. Aspects of the Stop TB strategy such as Public-Private Mix DOTS[89] aim to transition non-DOTS patients into DOTS programs, thus allowing them to benefit from higher DOTS treatment success rates and free care. This represents a positive development. However, it is crucial to distinguish between such activities—improvements in the care of patients already detected—and the detection of truly new cases. The new Stop TB strategy contains some provisions for increasing true case detection,[90] but there is no indication that empirical evaluations of these strategies have been performed or planned, raising the possibility that they may not have the desired impact. Screening for tuberculosis in the context of HIV services, for example, is limited by the fact that most tuberculosis is estimated to occur in patients without HIV co-infection.[3] In the absence of clear evidence on the effectiveness of existing techniques, broadly-applicable new strategies for actively detecting new cases are urgently needed.

## Supporting Information

Technical Appendix S1 Further details regarding data sources and methods.
(0.07 MB DOC)

Table S1 Smear-positive notification rate as a function of GDP, HIV, and DOTS programme variables, 1995–2005: Sensitivity analysis of different functional forms and temporal configurations of lagged variables
(0.10 MB DOC)

Table S2 Smear-positive notification rate as a function of GDP, HIV, DOTS programme, DTP3 coverage, and population-related control variables, 1995–2005
(0.13 MB DOC)

Table S3 Smear-positive notification rate as a function of GDP, HIV, and education- and smoking-related control variables, 1995–2005
(0.11 MB DOC)

Table S4 Mean treatment success rate as a function of GDP, HIV, and DOTS coverage, 1995–2004. Sensitivity analysis without lagged dependent variable
(0.06 MB DOC)
