# Supplementary material for: Has the DOTS Strategy Improved Case Finding or Treatment Success? An Empirical Assessment
Source: PLoS One. 2008 Mar 5;3(3):e1721. doi: 10.1371/journal.pone.0001721 (PMC2253827; doi:10.1371/journal.pone.0001721)
Supplement: Technical Appendix S1 — Further details regarding data sources and methods. (0.07 MB DOC) [file pone.0001721.s001.doc]

**Technical appendix S1: Has the DOTS Strategy Improved Case Finding or Treatment Success? An Empirical Assessment**

Ziad Obermeyer^1,2*^, Jesse Abbott-Klafter^3^, Christopher JL Murray^2^

**1** Harvard Medical School, Boston, MA, United States of America, **2** Institute for Health Metrics and Evaluation, University of Washington, Seattle, WA, United States of America, **3** University of California at San Francisco School of Medicine, San Francisco, CA, United States of America

* To whom correspondence should be addressed. E-mail: ziad_obermeyer@hms.harvard.edu

**Methods**

Our statistical methods are standard econometric techniques for the analysis of time series cross-sectional data. The properties of these data, most notably their dynamic nature and the existence of repeated observations from the same panel (in our case, country), call for specific analytical methods to minimize the likelihood of incorrect inferences.

*Model*. Our analytic method takes advantage of the relationship between case notification rate (CNR), defined as the ratio of notified cases to population, and case detection rate, whereby changes in notifications are driven by changes in detection or changes in incidence rate (IR).

*CNR = CDR ∙ IR*

Since this multiplicative equation is the basis for our regression model, and since the independent variables occasionally have zero values, we choose the log-linear functional form as most logical and parsimonious way to capture the relationship. In other words, the following equation must also hold true:

$$LnCNR_{it}=\beta D_{it}+\gamma T_{it}+\varepsilon_{it}$$

Where *D_it_* is a vector of the determinants of variation in CDR, including variables pertaining to DOTS programmes, for country *i* in year *t*, and *T_it_* is a vector of determinants of incidence rate. We also present results in Table A1 for two other functional forms: one in which the CNR variable is not log-transformed, and one in which the dependent variable is the change in the CNR from the previous year. Results are similar in all three versions.

*Lagged variables*. The inclusion of a lagged dependent variable (LDV) is one of the most powerful methodological developments in time-series cross-sectional analysis, and is commonly applied to serially autocorrelated data.[1–5] The net effect of including a LDV is to reduce the omitted-variable bias pervasive in cross-sectional analyses. For example, more developed countries are likely to have stronger pre-existing health systems; they may thus be both better-equipped to roll out new health interventions or programmes like DOTS, and more likely to have lower overall tuberculosis incidence. In these cases, an analysis without a LDV is likely to yield a spurious negative coefficient on programme variables, when in fact other unobserved factors are at work.

We apply the Wooldridge test for serial autocorrelation[5] to all data used in this study. The presence of serial autocorrelation is an indication that the dependent variable is characterised by persistent or mean-reverting dynamics, implying that omitted variables are having a large impact on the dependent variable. When the Wooldridge test indicates autocorrelation, we include the one-year lag of the dependent variable in the model to control for these dynamics. In practice, this technique captures the cumulative effect of multiple unobserved variables from year to year—for example, a specific dynamic characteristic of the tuberculosis epidemic, health system performance, or the data recording and reporting process—in individual countries, and enables the effect of the remaining independent variables to be more directly estimated.

In models with SSNR as the dependent variable, we also test one-year lagged programme variables and different temporal configurations of the lagged HIV prevalence term, as independent variables, to capture different possible temporal relationships with case notifications.

*Fixed effects*. Every country is likely to have a unique set of time-invariant characteristics that are strong determinants of tuberculosis incidence and case detection, but unnecessary or impractical to include in a model. Some examples of these factors are climate, geographical features, stable national governance or health system structures, and others. Practically speaking, country fixed effects are a set of independent dummy variables, *a_i_*: (*i* = 1, …, *N*, where *N* is the number of countries in the study): *a_i_* = 1 if a given country-year observation comes from country *i*, and *a_i_* = 0 if it comes from another country.

In general we believe that the inclusion of fixed effects is justified for two reasons. First, on a theoretical level, it is likely that time-invariant determinants of treatment success and case notifications exist in individual countries. These can pertain to overall health system and programme quality, or underlying characteristics of the tuberculosis epidemic. Secondly and more empirically, we tested models with and without fixed effects. The LDV coefficients, typically just under one in models without fixed effects, are typically reduced by one-half to one-third with the addition of effects. Since the model *R^2^* does not decrease as a consequence of this change, one year’s value is no longer such a strong predictor of the subsequent year’s value. This gives more explanatory power to the remaining independent variables and increases our confidence in the parameter estimates. Indeed, in models exploring treatment success, addition of effects decreased the lag coefficient’s magnitude by one-third and made it non-significant, while increasing the model’s *R^2^*; it also tripled the magnitude of the DOTS effect on treatment success.

*Clustered standard errors*. Calculating standard errors clustered by country addresses the issue of non-independence of standard errors, common in datasets with repeated observations from the same cluster (in our case, country). In our dataset, assuming that all standard errors are random across the variance-covariance matrix may bias the significance of the results (though the magnitude of coefficients would not be affected): standard errors from the same country are likely to be correlated, typically leading to falsely low estimates of standard errors. Calculating standard errors clustered by country makes regression results robust to this correlation, as well as to arbitrary heteroskedasticity.

*Data sources*

| **Series** | **Source** |
| --- | --- |
| Case notifications | *GTBC* 2003-2007, *GAID* |
| Treatment success rate | *GTBC* 2003-2007 |
| DOTS component variables | *GTBC* 2003-2007 |
| GDP | Penn World Tables |
| HIV prevalence | UNAIDS |
| DTP3 coverage | UNICEF/WHO |
| Health expenditure per head | World Bank |
| Literacy rates | UNESCO |
| Total years of schooling | Barro-Lee estimates |
| Urban population | UN Population Division |
| Smoking impact ratio | WHO |
| Apparent cigarette consumption | American Cancer Society |
| Population | UN Population Division |

Notes:

- - - - *DOTS component variables:* We test as independent variables (both individually and in groups) the following country-level indicators of the activities of DOTS programmes at the country level, all of which are reported by WHO in their GTBC reports.
      - The presence of directly-observed therapy
      - Existence of a high-quality drug supply chain
      - Presence of short-course chemotherapy
      - Existence of a national tuberculosis control manual
      - Training for national tuberculosis programme staff
      - Presence of facilities offering diagnosis by sputum microscopy
      - Comprehensive outcomes monitoring for cases on treatment
      - Existence of systems for follow-up of patients suspected of having tuberculosis but not diagnosed

None had any significant effect on treatment success or case notifications, nor did including them change the magnitude or significance of the DOTS independent variables (not shown).

- *GTBC*: Global Tuberculosis Control Report series, published by WHO. References in manuscript.
- *GAID*: Global Atlas of Infectious Diseases, maintained by WHO. http://globalatlas.who.int (accessed 23 June, 2007).
- *Barro-Lee Educational Attainment*: Barro, Robert J. and Jong-Wha Lee, "[International Data on Educational Attainment: Updates and Implications](http://www.cid.harvard.edu/cidwp/042.htm)" (CID Working Paper No. 42, April 2000). http://www.cid.harvard.edu/ciddata/ciddata.html (accessed May 27, 2006).

*Electronic reporting of case notifications*. We perform a literature review (PubMed, Sociofile, Google Scholar, WHO website; July 2007) to identify countries with any indication of a change in reporting modality for tuberculosis cases over the time period of our analysis. Specific examples include transitions to an internet-based reporting system, or an electronic register of tuberculosis cases which was transmitted digitally to the central reporting mechanism. We conservatively do not include countries that implementing a more general district health information system not specifically pertaining to tuberculosis cases.

**Discussion**

*Table S1*

*Concurrent-year HIV prevalence*. We use a lagged HIV prevalence variable to account for the period during which significant immune compromise accelerates breakdown from latent tuberculosis infection to clinical disease. Since the actual time-dependence of tuberculosis notifications on HIV prevalence is unknown, we test a wide variety of temporal configurations. In the main text, we present results using the five-year-lagged HIV prevalence; we here present results using a concurrent-year HIV prevalence variable, as an example of a different specification. The coefficients in these models are largely similar to those in the models using five-year-lagged HIV prevalence, as well as the seven- and ten-year-lagged values (not shown). One exception is that country effects make HIV non-significant in both models using concurrent-year HIV prevalence, but only in one model (Model 1) with five year-lagged HIV prevalence.

*One-year lagged programme variables*. It is possible that programme changes affect notifications in the subsequent year, not the concurrent year, and we test one-year-lagged independent programme variables to rule out such a delayed impact. The coefficients are similar to those models using concurrent-year independent programme variables, reassuring us that our results are stable.

*SSNR not log-transformed*. While the choice of log-linear functional form for models exploring change in case notification rates is based on our understanding of the relationship among notifications, detection, and incidence, other relationships are possible. The most obvious is a simple linear model, the results of which we present here. The coefficients are stable across a range of functional forms.

*Differences-in-differences model*. Another rigorous functional form commonly used in time-series cross-sectional analysis is the differences-on-differences, or first-differenced, model. This model regresses year-to-year change in the dependent variable on year-to-year change in the independent variables. Coefficients are similar in sign and significance to the models presented in the main text.

*Tables S2 and S3*

These tables present the results of models exploring case notifications, in which we test number of control variables, pertaining to both case detection and tuberculosis incidence. Our approach is to first to test these variables in models alone with a LDV, GDP and HIV, with or without fixed effects. We subsequently add the control variable to models including DOTS programme variables, in order to observe whether it changes the magnitude or significance of the DOTS coefficients. In these tables, we present the results of the models testing the variables alone; when the variable is found to be consistently significant, we also present results from models in which the control variable is included along with DOTS programme variables. Overall, no variable is found to be consistently significant except for urbanization, and none impact the DOTS coefficients.

*DTP3 coverage*. Coverage of DTP3, three doses of diphtheria, tetanus, and pertussis vaccination, can be used as an indicator of health national system performance, as it represents the ability of the system to deliver a basic intervention. We include this in some models to control for the possibility that broader health system change, unrelated to DOTS programmes, could impact notifications. Inclusion of DTP3 does not affect the magnitude or significance of the DOTS variables. The negative, significant coefficient seen in models without fixed effects disappears with their addition. This may represent a time-invariant correlation between better health system performance and lower levels of incident tuberculosis.

*Percent of population over 65 years old*. We might expect an aging population to create more incident tuberculosis as a result of increased breakdown; alternatively, since aging populations are more common in more developed countries with less incident tuberculosis, we might also observe a negative relationship between the two variables. We test the effect of demographic composition by including a variable measuring the percent of a country’s population over 65 years old in a given year. This is non-significant, and has no impact on the coefficients of the DOTS variables. This could be because countries with the most tuberculosis have fewer individuals over 65, or because the two effects offset each other.

*Percent of population living in urban areas*. There are two major ways in which rates of urbanisation could affect case notifications. First, increased population density and other aspects of the urban environment (sunlight exposure, air quality, *etc*.) could drive increased incident tuberculosis. Second, increased population density could also make it easier for tuberculosis programmes to detect incident cases by concentrating vulnerable populations in smaller geographical areas. This variable is significant and positive in models with and without fixed effects; its magnitude is increased by the fixed effects. Our model does not allow us to distinguish between the two mechanisms by which urbanization might increase notifications. Including urbanization in models with independent DOTS programme variables has no impact on those coefficients, with or without (not shown) fixed effects.

*Education*. Education could function as a measure of general development and thus be correlated with reduced tuberculosis incidence; alternatively, it could act directly to increase case detection by increasing the likelihood of self-presentation to a health facility. We test two measures of educational achievement in our models (Barro-Lee estimates of total years of schooling and overall population literacy rates), neither of which has any impact on notifications. This could be because the effect sizes are small, or because the two effects tend to counterbalance each other.

*Smoking*. There is increasing evidence that smoking may predispose to the development of active tuberculosis, either by increasing the annual risk of infection or increasing the probability of breakdown. We test two measures of smoking prevalence. First, the Smoking Impact Ratio (SIR), used in WHO’s most recent Global Burden of Disease figures, estimates the impact of smoking by subtracting death rates from non-smoking-related lung cancers from death rates from smoking-related cancers. Similar to HIV, the time-dependence of tuberculosis notifications on smoking is unknown, and we thus test a number of different temporal configurations. Second, data on apparent cigarette consumption uses the overall national consumption of cigarettes to measure rates of smoking. In none of these models do we observe a significant impact of smoking on case notifications. This may be due to the fact that the epidemiological impact is not large, or may be due to the limitations of the data assessing smoking prevalence.

*Table S4*

In this table we present results from two alternate models exploring treatment success rates.

*Models without a lagged dependent variable*. Though the Wooldridge test indicates serial autocorrelation (*p* = 0·0002), the LDV coefficient is not significant in the models presented in the main text. There is thus the possibility that models without a LDV are also appropriate ways to analyse these data. We retest both models without a LDV, and observe that the magnitude and significance of the programme coefficients is robust to this change.
