# Supplementary material for: Has the DOTS Strategy Improved Case Finding or Treatment Success? An Empirical Assessment
Source: PLoS One. 2008 Mar 5;3(3):e1721. doi: 10.1371/journal.pone.0001721 (PMC2253827; doi:10.1371/journal.pone.0001721)
Supplement: Table S1 — Smear-positive notification rate as a function of GDP, HIV, and DOTS programme variables, 1995–2005: Sensitivity analysis of different functional forms and temporal configurations of lagged variables (0.10 MB DOC) [file pone.0001721.s002.doc]

|  |  | **Panel regressions** | | | | | | | |  | **Differences-in-**  **differences^†^** | |
| --- | --- | --- | --- | --- | --- | --- | --- | --- | --- | --- | --- | --- |
|  |  | Concurrent-year HIV | |  | 1yr-lagged programme variables | |  | SSNR not log-transformed | |  |
|  |  | *Model 1* | *Model 2* |  | *Model 1* | *Model 2* |  | *Model 1* | *Model 2* |  | *Model 1* | *Model 2* |
| ***GDP per head,*** | *Coefficient* | 0.009 | 0.007 |  | 0.002 | **0.024** |  | 0.008 | 0.006 |  | -0.018 | **-0.071** |
| ***USD thousands*** | *SE* | 0.008 | 0.014 |  | 0.006 | 0.009 |  | 0.008 | 0.013 |  | 0.021 | 0.019 |
|  |  |  |  |  |  |  |  |  |  |  |  |  |
| ***HIV seroprevalence*** | *Coefficient* | -0.11 | 1.60 |  | 0.44 | **0.93** |  | 0.45 | **1.40** |  | **5.70** | **3.14** |
| *(5-yr lag unless noted)* | *SE* | 1.50 | 0.84 |  | 0.41 | 0.47 |  | 0.52 | 0.53 |  | 2.24 | 0.68 |
|  |  |  |  |  |  |  |  |  |  |  |  |  |
| ***DOTS population*** | *Coefficient* | 0.06 | - |  | 0.07 | - |  | 0.05 | - |  | 0.02 | - |
| ***coverage fraction*** | *SE* | 0.05 |  |  | 0.04 |  |  | 0.05 |  |  | 0.04 |  |
|  |  |  |  |  |  |  |  |  |  |  |  |  |
| ***DOTS treatment*** | *Coefficient* | - | 0.06 |  | - | 0.11 |  | - | 0.04 |  | - | -0.26 |
| ***success fraction*** | *SE* |  | 0.12 |  |  | 0.13 |  |  | 0.12 |  |  | 0.14 |
|  |  |  |  |  |  |  |  |  |  |  |  |  |
| ***Lag of SSNR*** | *Coefficient* | **0.56** | **0.50** |  | **0.62** | **0.61** |  | **0.56** | **0.49** |  | - | - |
| *(one year)* | *SE* | 0.05 | 0.05 |  | 0.05 | 0.06 |  | 0.05 | 0.05 |  |  |  |
|  |  |  |  |  |  |  |  |  |  |  |  |  |
| ***Constant*** | *Coefficient* | **0.67** | **1.09** |  | **0.75** | **1.11** |  | **0.69** | **1.14** |  | **0.03** | **0.04** |
|  | *SE* | 0.27 | 0.13 |  | 0.24 | 0.25 |  | 0.27 | 0.14 |  | 0.01 | 0.01 |
|  |  |  |  |  |  |  |  |  |  |  |  |  |
| ***Observations (country-years)*** | | 1128 | 887 |  | 1029 | 938 |  | 1128 | 887 |  | 1015 | 783 |
| ***R^2^*** |  | 0.95 | 0.96 |  | 0.96 | 0.96 |  | 0.95 | 0.96 |  | 0.01 | 0.02 |
|  |  |  |  |  |  |  |  |  |  |  |  |  |
| Coefficients significant at the 0.05 level are in bold. All standard errors are clustered by country. | | | | | | |  |  |  |  |  |  |
|  |  |  |  |  |  |  |  |  |  |  |  |  |
| ***Table S1: Smear-positive notification rate as a function of GDP, HIV, and DOTS programme variables, 1995-2005*** | | | | | | | | | |  |  |  |
| ***Sensitivity analysis of different functional forms and temporal configurations of lagged variables*** | | | | | | | | |  |  |  |  |
| *(Independent programme variables: Model 1*—*DOTS population coverage, Model 2—DOTS treatment success rate)* | | | | | | | | | | | | |
| *^†^1996-2005* | | | |  |  |  |  |  |  |  |  |  |
