# Supplementary material for: Has the DOTS Strategy Improved Case Finding or Treatment Success? An Empirical Assessment
Source: PLoS One. 2008 Mar 5;3(3):e1721. doi: 10.1371/journal.pone.0001721 (PMC2253827; doi:10.1371/journal.pone.0001721)
Supplement: Table S2 — Smear-positive notification rate as a function of GDP, HIV, DOTS programme, DTP3 coverage, and population-related control variables, 1995–2005 (0.13 MB DOC) [file pone.0001721.s003.doc]

|  |  | **DTP3 coverage control variable** | | | |  | **Population structure-related control variables** | | | | | |
| --- | --- | --- | --- | --- | --- | --- | --- | --- | --- | --- | --- | --- |
|  |  | Alone |  | With programme variables | |  | % pop >65 years old |  | % pop urban |  | % pop urban, with programme variables | |
|  |  |  |  | *Model 1* | *Model 2* |  |  |  |  |  | *Model 1* | *Model 2* |
| ***GDP per head,*** | *Coefficient* | -0.0004 |  | 0.0062 | 0.0051 |  | 0.0006 |  | -0.0022 |  | 0.0042 | -0.0055 |
| ***USD thousands*** | *SE* | 0.0095 |  | 0.0099 | 0.0134 |  | 0.0137 |  | 0.0054 |  | 0.0076 | 0.0082 |
|  |  |  |  |  |  |  |  |  |  |  |  |  |
| ***HIV seroprevalence*** | *Coefficient* | 1.14 |  | 0.68 | **1.39** |  | **1.21** |  | 0.49 |  | 0.20 | 0.93 |
| ***(five-year lag)*** | *SE* | 0.61 |  | 0.63 | 0.53 |  | 0.61 |  | 0.54 |  | 0.57 | 0.60 |
|  |  |  |  |  |  |  |  |  |  |  |  |  |
| ***DOTS population*** | *Coefficient* | - |  | 0.04 | - |  | - |  | - |  | 0.04 | - |
| ***coverage fraction*** | *SE* |  |  | 0.05 |  |  |  |  |  |  | 0.05 |  |
|  |  |  |  |  |  |  |  |  |  |  |  |  |
| ***DOTS treatment*** | *Coefficient* | - |  | - | 0.03 |  | - |  | - |  | - | -0.05 |
| ***success fraction*** | *SE* |  |  |  | 0.12 |  |  |  |  |  |  | 0.12 |
|  |  |  |  |  |  |  |  |  |  |  |  |  |
| ***DTP3 coverage*** | *Coefficient* | 0.19 |  | 0.16 | 0.06 |  | - |  | - |  | - | - |
| ***fraction*** | *SE* | 0.13 |  | 0.13 | 0.11 |  |  |  |  |  |  |  |
|  |  |  |  |  |  |  |  |  |  |  |  |  |
| ***Percent population*** | *Coefficient* | - |  | - | - |  | - |  | **0.016** |  | 0.012 | **0.021** |
| ***urban*** | *SE* |  |  |  |  |  |  |  | 0.007 |  | 0.008 | 0.010 |
|  |  |  |  |  |  |  |  |  |  |  |  |  |
| ***Percent population*** | *Coefficient* | - |  | - | - |  | 0.005 |  | - |  | - | - |
| ***over 65 years old*** | *SE* |  |  |  |  |  | 0.036 |  |  |  |  |  |
|  |  |  |  |  |  |  |  |  |  |  |  |  |
| ***Lag of SSNR*** | *Coefficient* | **0.49** |  | **0.49** | **0.49** |  | **0.49** |  | **0.54** |  | **0.55** | **0.47** |
| ***(one year)*** | *SE* | 0.04 |  | 0.05 | 0.05 |  | 0.04 |  | 0.04 |  | 0.05 | 0.05 |
|  |  |  |  |  |  |  |  |  |  |  |  |  |
| ***Constant*** | *Coefficient* | **1.94** |  | **2.40** | **1.10** |  | **1.61** |  | -0.35 |  | **0.91** | -0.04 |
|  | *SE* | 0.18 |  | 0.18 | 0.15 |  | 0.53 |  | 0.73 |  | 0.25 | 0.65 |
|  |  |  |  |  |  |  |  |  |  |  |  |  |
| ***Observations(country-years)*** | | 1120 |  | 1007 | 884 |  | 1108 |  | 1241 |  | 1128 | 887 |
| ***R^2^*** |  | 0.94 |  | 0.96 | 0.96 |  | 0.95 |  | 0.95 |  | 0.96 | 0.96 |
|  |  |  |  |  |  |  |  |  |  |  |  |  |
| Coefficients significant at the 0.05 level are in bold. All standard errors are clustered by country. | | | | | | | |  |  |  |  |  |
|  |  |  |  |  |  |  |  |  |  |  |  |  |
| ***Table S2: Smear-positive notification rate as a function of GDP, HIV, DOTS programme, DTP3 coverage, and population-related control variables, 1995-2005*** | | | | | | | | | | | | |
| *(Independent programme variables: Model 1*—*DOTS population coverage, Model 2—DOTS treatment success rate)* | | | | | | | | | | | | |
