# Supplementary material for: Has the DOTS Strategy Improved Case Finding or Treatment Success? An Empirical Assessment
Source: PLoS One. 2008 Mar 5;3(3):e1721. doi: 10.1371/journal.pone.0001721 (PMC2253827; doi:10.1371/journal.pone.0001721)
Supplement: Table S3 — Smear-positive notification rate as a function of GDP, HIV, and education- and smoking-related control variables, 1995–2005 (0.11 MB DOC) [file pone.0001721.s004.doc]

|  |  | **Education control variables** | | |  | **Smoking control variables** | | | | |
| --- | --- | --- | --- | --- | --- | --- | --- | --- | --- | --- |
|  |  | Total school |  |  |  | Smoking impact ratio | | |  | Cigarette |
|  |  | years |  | Literacy rates |  | *Impact ratio lag years* | | |  | consumption |
|  |  |  |  |  |  | concurrent | 10 | 20 |  |  |
| ***GDP per head,*** | *Coefficient* | -0.004 |  | 0.003 |  | **-0.055** | **-0.009** | 0.002 |  | -0.027 |
| ***USD thousands*** | *SE* | 0.010 |  | 0.015 |  | 0.020 | 0.004 | 0.009 |  | 0.037 |
|  |  |  |  |  |  |  |  |  |  |  |
| ***HIV seroprevalence*** | *Coefficient* | 0.98 |  | 0.30 |  | 20.50 | 13.58 | 11.22 |  | 3.25 |
| ***(five-year lag)*** | *SE* | 0.65 |  | 0.58 |  | 49.02 | 13.65 | 16.37 |  | 1.77 |
|  |  |  |  |  |  |  |  |  |  |  |
| ***Average*** | *Coefficient* | - |  | 0.0103 |  | - | - | - |  | - |
| ***literacy rate*** | *SE* |  |  | 0.0079 |  |  |  |  |  |  |
|  |  |  |  |  |  |  |  |  |  |  |
| ***Total school*** | *Coefficient* | 0.04 |  |  |  | - | - | - |  | - |
| ***years expected*** | *SE* | 0.07 |  |  |  |  |  |  |  |  |
|  |  |  |  |  |  |  |  |  |  |  |
| ***Smoking impact*** | *Coefficient* | - |  | - |  | -0.0017 | -0.0018 | -0.0008 |  | - |
| ***ratio*** | *SE* |  |  |  |  | 0.0028 | 0.0020 | 0.0019 |  |  |
|  |  |  |  |  |  |  |  |  |  |  |
| ***Cigarette consumption*** | *Coefficient* | - |  | - |  | - | - | - |  | 0.43 |
| ***per head (thousands)*** | *SE* |  |  |  |  |  |  |  |  | 0.47 |
|  |  |  |  |  |  |  |  |  |  |  |
| ***Lag of SSNR*** | *Coefficient* | **0.47** |  | **0.47** |  | **0.36** | **0.49** | **0.39** |  | **0.28** |
| ***(one year)*** | *SE* | 0.06 |  | 0.05 |  | 0.11 | 0.08 | 0.10 |  | 0.06 |
|  |  |  |  |  |  |  |  |  |  |  |
| ***Constant*** | *Coefficient* | **2.08** |  | 0.23 |  | 2.51 | **1.71** | **1.96** |  | **1.49** |
|  | *SE* | 0.23 |  | 0.89 |  | 1.38 | 0.73 | 0.66 |  | 0.22 |
|  |  |  |  |  |  |  |  |  |  |  |
| ***Observations(country-years)*** | | 713 |  | 837 |  | 139 | 307 | 252 |  | 513 |
| ***R^2^*** |  | 0.94 |  | 0.95 |  | 0.85 | 0.87 | 0.93 |  | 0.94 |
|  |  |  |  |  |  |  |  |  |  |  |
| Coefficients significant at the 0.05 level are in bold. All standard errors are clustered by country. | | | | | | |  |  |  |  |
|  |  |  |  |  |  |  |  |  |  |  |
| ***Table S3: Smear-positive notification rate as a function of GDP, HIV, and education- and smoking-related control variables, 1995-2005*** | | | | | | | | | | |
