# Supplementary material for: Has the DOTS Strategy Improved Case Finding or Treatment Success? An Empirical Assessment
Source: PLoS One. 2008 Mar 5;3(3):e1721. doi: 10.1371/journal.pone.0001721 (PMC2253827; doi:10.1371/journal.pone.0001721)
Supplement: Table S4 — Mean treatment success rate as a function of GDP, HIV, and DOTS coverage, 1995–2004. Sensitivity analysis without lagged dependent variable (0.06 MB DOC) [file pone.0001721.s005.doc]

|  |  |  |  |  |
| --- | --- | --- | --- | --- |
|  |  |  |  |  |
|  |  |  |  |  |
|  |  |  | **With HIV** | **Without HIV** |
| ***GDP per head,*** | *Coefficient* |  | 0.02 | 0.02 |
| ***USD thousands*** | *SE* |  | 0.02 | 0.01 |
|  |  |  |  |  |
| ***HIV seroprevalence*** | *Coefficient* |  | -0.21 | - |
|  | *SE* |  | 0.50 |  |
|  |  |  |  |  |
| ***DOTS population*** | *Coefficient* |  | **0.17** | **0.16** |
| ***coverage fraction*** | *SE* |  | 0.08 | 0.06 |
|  |  |  |  |  |
| ***Constant*** | *Coefficient* |  | **0.50** | -0.12 |
|  | *SE* |  | 0.06 | 0.30 |
|  |  |  |  |  |
| ***Observations(country-years)*** |  |  | 222 | 268 |
| ***R^2^*** |  |  | 0.72 | 0.74 |
|  |  |  |  |  |
| Coefficients significant at the 0.05 level are in bold. All standard errors clustered by country. | | | | |
|  |  |  |  |  |
| ***Table S4: Mean treatment success rate as a function of GDP, HIV, and DOTS coverage, 1995-2004. Sensitivity analysis without lagged dependent variable*** | | | | |
